# Capability beliefs on, and use of evidence-based practice among four health professional and student groups in geriatric care: A cross sectional study

**DOI:** 10.1371/journal.pone.0192017

**Published:** 2018-02-14

**Authors:** Anne-Marie Boström, Disa K. Sommerfeld, Annika W. Stenhols, Anna Kiessling

**Affiliations:** 1 Department of Neurobiology, Care Sciences and Society, Division of Nursing, Karolinska Institutet, Huddinge, Sweden; 2 Theme Aging, Karolinska University Hospital, Huddinge, Sweden; 3 Department of Nursing, Western Norway University of Applied Sciences, Campus Haugesund, Norway; 4 University Department of Rehabilitation Medicine, Danderyd Hospital, Danderyd, Sweden; 5 Department of Neurobiology, Care Sciences and Society, Division of Physiotherapy, Karolinska Institutet, Huddinge, Sweden; 6 Division of Orthopaedics, Danderyd Hospital, Danderyd, Sweden; 7 Department of Clinical Sciences Danderyd Hospital, Karolinska Institutet, Stockholm, Sweden; Kyoto University, JAPAN

## Abstract

Implementation of evidence-based practice (EBP) is a complex task. This study, conducted in an acute geriatric setting, aims to compare self-reported capability beliefs on EBP between health professionals and students, and to compare the use of EBP between health professional groups. Occupational therapists, physicians, physiotherapists and registered nurses with three or more months’ employment, and all students from the occupational therapy, medical, physiotherapy and nursing programs, who had conducted workplace learning at the department, were invited. Data on capability beliefs and use of EBP were collected using the Evidence-based Practice Capabilities Beliefs Scale assessing six activities of EBP: *formulate questions; search databases; search other sources; appraise research reports; participate in implementation in practice;* and *participate in evaluation*. Descriptive and inferential statistics were used. Capability beliefs on EBP: The health professionals (n = 101; response rate 80%) reported high on *search other sources* but less on *appraise research reports*. The students (n = 124; response rate 73%) reported high on all EBP activities. The health professionals reported significantly higher on *search other sources* than the students. The students reported significantly higher on *formulate questions* and *appraise research reports* than the health professionals. No significant differences were identified between the health professional groups or between the student groups. Use of EBP: Health professionals reported wide-ranging use from several times each month to once every six months. The physicians reported significantly more frequent use than registered nurses and occupational therapists. Health professionals supervising students reported more frequent use of *appraise research reports* than the non-supervising group. There is a need for improving the use of EBP, particularly among registered nurses and occupational therapists. Supervision of students might enhance the motivation among staff to increase the use of EBP and students’ high EBP capability beliefs might inspire staff in this matter.

## Introduction

Implementation of evidence-based guidelines, i.e. evidence-based recommendations, into evidence-based practice (EBP) is a complex task. A marked gap exists between what is done and what should be done to maximize patients’ health outcome. In 2003, Grol and Grimshaw reported that 30–40% of patients do not receive EBP, and that 20–25% of given treatments may be unnecessary or even harmful [1]. Also in the year 2003, the Institute of Medicine published the Health Professions Education: A Bridge to Quality report describing five generic competencies needed to achieve a high standard of health care: *All health professionals should be educated to deliver patient-centered care as members of an interdisciplinary team*, *emphasizing evidence-based practice*, *quality improvement approaches*, *and informatics* (page 45, [2]). During the last decade, several studies have been published on health professionals’ knowledge, attitudes and skills to apply EBP and the use of EBP in practice revealing that the gap between what is done and what should be done still exists [3–5]. Different individual and organisational barriers of implementation have been proposed, such as lack of motivation, lack of EBP knowledge, heavy workload, other staff/management not supportive of EBP, lack of resources, lack of authority to change practice and workplace culture resistant to change [6–8]. Known facilitators include audit and feedback, local opinion leaders, and support from managers and colleagues [6,9,10].

The conceptual framework Promoting Action on Research Implementation in Health Services (PARIHS) was introduced in 1998, and has been revised recently to increase knowledge on how a successful implementation of research into routine practice can be understood [11]. In this framework four constructs have to be considered: the quality and type of evidence, the characteristics of the context, the recipients, and the way in which the implementation of evidence is facilitated. The construct recipient includes characteristics as skills and knowledge, values and beliefs, motivation and goals, and is now described in the revised PARIHS framework. Boström and coworkers [12] have identified that the individual factor “capability beliefs to apply EBP in clinical practice” is strongly associated with self-reported use of EBP. The Evidence-based Practice Capabilities Beliefs Scale (EBPCBS) has been validated to measure the capability beliefs to apply the six activities in the EBP process according to the Bandura method to measure self-efficacy [13]. The term self-efficacy is defined as: "confidence in their ability to succeed in specific situations" and a synonym to this term is capability belief [14,15]. According to Bandura, people's beliefs in their ability to perform the activity improve over time by practicing the activity in a supportive and stress-free environment [14,15].

A promoting health care organisation and leadership is crucial to achieve EBP. The importance of leadership is reported in several systematic reviews [16,17]. A study of Dannapfel and coworkers [18] also report that there is a leadership and managerial responsibility as to how well physiotherapists manage to increase the use of EBP in their clinical practice. Another Swedish study conducted in a national sample of registered nurses working in various health care settings, including geriatric settings found that the following organizational factors were positively associated with the use of EBP: supportive leadership, supportive team work (collective efficacy), increased job demands, control over work situation and working in the care of older people [12].

Studies have also evaluated students’ views on educational support needed to develop competence in EBP. Numerous of them have shown that occupational therapist, medical, physiotherapist and nursing students do not always feel supported to develop capability to apply EBP during their undergraduate education [19–23]. In summary, these studies show that students feelings of support may depend on pedagogic methods used such as workshop; supervisors’ attitudes towards EBP; access to role models in EBP; and the quality of the workplace culture (e.g. work satisfaction, hierarchies, work load, vacancies) where the students conduct workplace learning [19–23].

Different theories exist in the literature regarding professional competence development. Dall’Alba and Sandberg [24] have described it as a two-dimensional process: The gain of increased professional knowledge and skills; the gain of an embodied understanding of and in practice, i.e. an understanding of the context and culture where the professional skills are supposed to be applied. The way in which practice is understood forms and systematizes specific knowledge and skills into a separate competence dimension. To gain professional competence to conduct EBP in the context and culture in, e.g. an acute geriatric setting raises special challenges [25]. The patient population treated in hospital-based acute geriatric care is dominated by older patients with several comorbidities and complex health problems and needs.

Almost all evidence-based guidelines state evidence and recommendations based on studies of patients with a single disease. This guideline approach however results in a dilemma when health professionals encounter a patient with several comorbidities [26]. We argue that the difficulty in conducting EBP will increase even more when the patient is older. There are at least two reasons influencing this difficulty: Most of the science behind the evidence has been generated from randomized controlled studies on selected populations excluding patients over a certain age and often also excluding patients with comorbidities. Two systematic reviews scrutinizing the evidence-base for treatment to older adults illustrate these difficulties, and argues for more randomized controlled studies on older adults [27,28]. We further argue that the quality of care for the more complex patient mix in geriatric care will improve if they are cared for by staff, with a comprehensive mix of competencies, who work together in inter-professional teams. Several studies have reported improved outcomes for the older patient population in geriatric care settings using comprehensive geriatric assessments and teamwork [29]. In addition, interventions with person-centered care for older adults have, for example, improved the discharge processes [30]. This may be due to that patients were looked upon as enough competent to be involved in planning their subsequent care [30]. To achieve high standard of care for older adults in geriatric care, health professionals need to master and practice the generic competences suggested by the Institute of Medicine [2].

To conclude there is a solid scientific base on the gap between recommendations stated in the evidence-based guidelines and the care provided in routine care. Further, several barriers and facilitators influencing the implementation of EBP have been explored. However, the evidence is scarce on health professionals’ and students’ levels on capability beliefs to perform EBP. The aim of this study was twofold; to compare self-reported capability beliefs on EBP between health professional and student groups, and to compare the use of EBP between four health professional groups in a hospital-based acute geriatric care setting in Sweden.

## Material and methods

### Design

We used a cross-sectional correlational study design.

### Setting

The study was conducted in a geriatric department with 110 hospital beds for acute admittance, stroke rehabilitation, and orthopedic rehabilitation of older patients often with comorbidities. During the study period 255 persons were employed in total, of which approximately 150 were occupational therapists, physicians, physiotherapists and registered nurses. Each semester some 150 students from the undergraduate occupational therapy, medical, physiotherapy and nursing programs performed workplace learning at the department. Two clinical lecturers (Master prepared) and two senior lecturers (PhD degree) facilitated the students’ supervision and managed the students at the department during the workplace learning periods.

### Sample

In fall 2012 (September to December) we invited all occupational therapists, physicians, physiotherapists and registered nurses with three or more months employment (n = 127) and all students from the occupational therapy, medical, physiotherapy and nursing programs, who had workplace learning at the department (n = 81) to participate in the study. In fall 2013 (September to December), 88 additional students, i.e., those who had workplace learning during this period, were invited to participate in the study.

Inclusion criteria: occupational therapists, physicians, physiotherapists and registered nurses working in patient care and students from the occupational therapy, medical, physiotherapy and nursing programs. Exclusion criteria: less than three months employment for health professionals; and less than two weeks of workplace learning for students, except for those from the medical program who all had a period of one week (five days).

### Data collection

To collect data on self-reported capability beliefs of EBP the EBPCBS was used [13]. The EBPCBS is developed using the following definition of the EBP process: “From a defined question, seek out relevant knowledge, critical appraise and compile this knowledge, and implement the results of this appraisal in clinical practice” by Sackett and coworkers [31]. The six EBPCBS items reflect the EBP process as follows: 1) Formulating questions about clinical practice to search for new research-based knowledge (*formulate questions*), 2) using databases to search for knowledge (*search databases*), 3) using other information sources, e.g. books, journals or asking colleagues (*search other sources*), 4) appraising research reports (*appraise research reports*), 5) contributing to change in clinical practice by implementing research knowledge (*implement knowledge*), and 6) participating in evaluating whether clinical practice is based on research knowledge (*evaluate practice*). The respondents (health professionals and students) were asked to rate, on a scale from 0–3: No, I can’t manage that; 4 to 7: I might manage that; 8 to 10: I’m sure I can manage that, how confident they were about performing each and every step of the EBP process.

For health professionals, the EBPCBS also includes a section asking the respondents how often they carry out the EBP activities in their work. The respondents (health professionals) were asked to rate this on a scale from 1 to 4: 1 (seldom or never), 2 (about once every 6 months), 3 (about once a month) and 4 (several times a month). The EBP items and response options are presented in S1 Appendix.

Two indices were generated; one to measure the respondent’s capability beliefs on the whole EBP process, and another on the respondent’s use of the whole EBP process. The 6 items in the respective indices were summarized and divided by 6. The EBPCBS instrument has been developed in a national sample (n = 1256) of registered nurses working in various health care settings, including geriatric settings, and is considered valid and reliable using Rasch modelling [13]. It has been used in several studies among registered nurses working in different types of health care settings in Sweden, including geriatric settings [12,32–34] and among registered nurses, physical therapists, pharmacists and social workers working in an acute care hospital in the United States [35] and among nursing students from 26 universities and university colleges in Sweden [19].

Data were collected on the following demographic data for the health professionals: gender, age, years in profession, experience of supervising students, and pedagogic competence, i.e. formal pedagogic education. Data were collected on the following demographic data for the students: gender, age, university, program and semester.

### Data collection procedure

The survey to health professionals contained 18 items (6 demographic variables, 6 items on EBP capability beliefs and 6 items on EBP use). The survey to students contained 11 items (5 demographic variables and 6 items on EBP Capability beliefs). The surveys were printed on paper. Health professionals received the survey at their workplace and they were asked to hand it in two weeks later. The students received the survey at the start of their workplace-learning period and were asked to hand it in at the end of that period. Reminders were sent twice to non-responders.

### Data analysis

The students who had conducted more than half time of their entire program were categorized as senior students and the rest as novice students. The students with less than three weeks placement at the ward were categorized as having a short placement and the rest as having a long placement.

The internal consistency for the EBP indices was evaluated using Cronbach’s alpha test.

Descriptive statistics were used for frequencies and distributions. Inferential statistics were used to compare the health professionals and student groups. Student’s t-test was used to compare two groups, and ANOVA was used to compare four groups, using Bonferroni posthoc test. Pearson correlation coefficient test was used to assess relationships between EBP indices and years in profession. Statistical significance for all analyses was set at P<0.05. Statistical analyses were performed using IBM SPSS Statistics for Windows, version 23.0 (IBM SPSS Inc., Chicago, IL).

### Ethical considerations

All participants received an information letter, which informed them about the purpose of the study and that participation was voluntary. The surveys were coded and the code key was used only by the person who sent reminders to those participants who did not hand in their answer. The study was approved by the Regional Ethical Review Board in Stockholm (No. 2012/847-31/5).

## Results

The response rate was 80% for health professionals and 73% for students. Missing data for each of the items varied between 0 and 1 person (0–1.0%) for EBP Capability beliefs and 2 and 4 persons (2.0–4.0%) for EBP use for the health professionals group and between 2 and 3 persons (1.6–2.4%) for EBP Capability beliefs for the student group. The alpha value for the EBP Capability beliefs index among health professionals was 0.88 and for the students 0.90. The alpha value for the EBP use index among health professionals was 0.79. The demographics for the health professionals and the students are presented in Table 1.

**Table 1:** Descriptions of the health professionals and student samples.

	Total Health professionals (n = 127)	Health professionals				Total Students	Students			
		OT n = 15	Physicians n = 27	PT n = 18	RN n = 67	n = 169	OT n = 13	Medical n = 69	PT n = 17	Nursing n = 70
**Response rate**	101 (80%)	10 (67%)	24 (89%)	14 (78%)	53 (79%)	124 (73%)	11 (85%)	40 (58%)	16 (94%)	57 (81%)
**Age** m (SD)	42 (12)	39 (12)	41 (12)	48 (13)	42 (11)	26 (5)	26 (4)	25 (4)	25 (5)	27 (6)
**Gender**										
Women	92 (91%)	10 (100%)	18 (75%)	14 (100%)	50 (94%)	94 (76%)	11 (100%)	21 (53%)	12 (75%)	50 (88%)
**Years in profession** m (SD)	12 (11)	8 (9)	12 (11)	21 (14)	11 (10)					
**Experience of supervising students** (yes)	65 (64%)	4 (40%)	21 (88%)	9 (64%)	31 (58%)					
**Formal pedagogic education** (yes)	50 (50%)	7 (70%)	13 (54%)	8 (57%)	22 (42%)					
**Time as student in the program**										
Completed ≤50%						55 (44%)	5 (45%)	24 (60%)	4 (25%)	22 (39%)
Completed >50%						69 (56%)	6 (55%)	16 (40%)	12 (75%)	35 (61%)
**Workplace learning period**										
Short ≤3 weeks						54 (44%)	6 (55%)	40 (100%)	8 (50%)	
Long >3 weeks						70 (56%)	5 (45%)		8 (50%)	57 (100%)

Values are given as mean ± standard deviation: m (SD); or as number and percentage: n (%)

OT denotes Occupational Therapist; PT denotes Physiotherapist; RN denotes Registered Nurse.

### Health professionals’ and students’ ratings of capability beliefs on EBP

The health professional group rated a mean value of 7.6 on the EBP capability beliefs index and between 6.9 and 9.2 on the six EBP activities. They rated the highest mean value on the EBP activity *Search other sources* and the lowest mean value on the EBP activity *Appraise research reports* (Table 2). The only significant difference on capability beliefs between the health professional groups was that physiotherapists rated significantly higher mean value (8.4) than the physicians (6.5) on the EBP activity *Implement knowledge* (Table 2). No significant differences were found between those who had supervised students and those who had not (S1 Table).

**Table 2:** Health professionals’ and students’ capability beliefs on evidence-based practice.

	Health professionals				ANOVA P-value	Posthoc Bonferroni	Students				ANOVA P-value	Posthoc Bonferroni
	OT	Physician	PT	RN			OT	Medical	PT	Nursing		
**EBP capability beliefs index**	6.8 (1.4)	7.4 (1.6)	7.8 (2.0)	7.7 (1.7)	0.329		8.0 (1.6)	7.6 (1.6)	8.4 (1.1)	8.3 (1.4)	0.088	
**Formulate questions**	5.1 (2.5)	7.4 (1.9)	7.4 (2.5)	7.1 (2.3)	0.048	Phys > OT (p = 0.055)	8.1 (1.5)	7.8 (1.9)	8.8 (1.2)	8.2 (2.0)	0.423	
**Search databases**	7.8 (1.6)	7.7 (2.1)	7.3 (2.7)	8.1 (2.4)	0.640		8.1 (1.3)	8.0 (1.6)	8.2 (1.6)	8.6 (1.8)	0.499	
**Search other sources**	8.9 (0.9)	9.1 (1.2)	9.4 (1.0)	9.2 (1.4)	0.837		8.8 (1.5)	8.2 (1.7)	9.1 (0.9)	9.0 (1.4)	0.042	Nursing > Medical (p = 0.050)
**Appraise research reports**	5.1 (3.0)	6.8 (2.3)	7.2 (2.7)	7.2 (2.2)	0.088		8.0 (1.9)	7.7 (1.9)	8.5 (1.1)	8.5 (1.6)	0.183	
**Implement knowledge**	7.0 (1.8)	6.5 (2.7)	8.4 (1.6)	7.6 (2.0)	0.039	PT > Phys (p = 0.040)	7.2 (2.9)	6.7 (2.0)	7.6 (2.1)	7.8 (2.0)	0.079	
**Evaluate practice**	6.6 (2.1)	6.9 (2.1)	7.4 (2.8)	7.4 (2.1)	0.647		7.6 (2.6)	6.9 (2.1)	7.9 (1.9)	7.8 (2.0)	0.169	

Values are given as mean ± standard deviation (SD). The p-values are calculated by ANOVA.

EBP denotes evidence-based practice; OT denotes Occupational Therapist; PT denotes Physiotherapist; RN denotes Registered Nurse; Phys denotes Physician.

Response alternatives range from 0 (No, I can’t manage that) to 10 (I’m sure I can manage that).

The students rated a mean value of 8.0 on the EBP capability beliefs index and between 7.4 and 8.8 on the six EBP activities. They rated the highest mean value on the EBP activity *Search other sources* and the lowest mean value for the EBP activity *Implement knowledge* (Table 2). No significant differences were found on capability beliefs between the students from the four programs.

The only significant difference between senior and novice students was that senior students rated higher capability beliefs on *Search databases* than novice students (8.8 vs. 7.8; p = 0.001) (S2 Table). The only significant difference between students with a long clinical placement (three weeks or longer) and those with a short clinical placement was that those with a long clinical placement rated significantly higher on *Search other sources* (9.0 vs. 8.4; p = 0.014) (S2 Table).

The student group rated significantly higher capability beliefs than the health professional group on the EBP capability beliefs index, and the EBP activities *Formulate questions* and *Appraise research reports*. The health professional group rated significantly higher than the student group on the EBP activity *Search other sources* (Table 3).

**Table 3:** Comparison of reported capability beliefs on evidence-based practice between health professionals and students.

	Health professionals	Students	P-value
**EBP capability beliefs index**	7.6 (1.7)	8.0 (1.5)	0.031
**Formulate questions**	7.0 (2.3)	8.1 (1.9)	<0.001
**Search databases**	7.9 (2.3)	8.3 (1.7)	0.086
**Search other sources**	9.2 (1.3)	8.8 (1.5)	0.022
**Appraise research reports**	6.9 (2.4)	8.2 (1.7)	<0.001
**Implement knowledge**	7.4 (2.2)	7.4 (2.1)	0.898
**Evaluate practice**	7.2 (2.2)	7.5 (2.1)	0.297

Values are given as mean ± standard deviation (SD). The p-values are calculated by unpaired t-test between total health professionals and total student groups.

Response alternatives range from 0 (No, I can’t manage that) to 10 (I’m sure I can manage that).

EBP denotes evidence-based practice.

### Health professionals’ use of EBP

Health professionals reported most frequent use of the EBP activities *Search other sources* (m = 3.6), *Search databases* (m = 2.6), and *Implement knowledge* (m = 2.5). The least frequently used EBP activity was *Appraise research reports* (m = 1.7) (Table 4).

**Table 4:** Health professionals reported use of evidence-based practice.

	Health professionals	Occupational Therapist	Physician	Physiotherapist	Registered Nurse	P-value ANOVA	Posthoc Bonferroni
**EBP use index**	2.5 (0.7)	2.2 (0.5)	2.8 (0.6)	2.6 (0.8)	2.3 (0.6)	0.015	Phys > RN (p = 0.030)
**Formulate questions**	2.2 (1.0)	1.7 (0.7)	2.8 (1.1)	2.6 (0.8)	1.9 (1.0)	0.001	Phys > RN (p = 0.003); Phys > OT (p = 0.019)
**Search databases**	2.6 (1.1)	2.1 (1.0)	3.0 (1.0)	2.4 (1.1)	2.6 (1.1)	0.140	
**Search other sources**	3.6 (0.6)	3.6 (0.5)	4.0 (0.2)	3.8 (0.6)	3.5 (0.6)	0.004	Phys > RN (p = 0.003)
**Appraise research reports**	1.7 (0.9)	1.4 (0.7)	2.3 (0.8)	1.9 (1.0)	1.5 (0.8)	0.001	Phys > RN (p = 0.002) Phys > OT (p = 0.029)
**Implement knowledge**	2.5 (1.0)	2.4 (0.8)	2.4 (1.0)	2.8 (1.0)	2.5 (1.0)	0.689	
**Evaluate practice**	2.1 (1.0)	1.9 (0.9)	2.2 (1.0)	2.2 (1.2)	2.0 (1.0)	0.832	

Values are given as mean ± standard deviation (SD). The p-values are calculated by ANOVA between professional groups.

EBP denotes evidence-based practice; OT denotes Occupational Therapist; PT denotes Physiotherapist; RN denotes Registered Nurse; Phys denotes Physician.

OT denotes Occupational Therapist; PT denotes Physiotherapist; RN denotes Registered Nurse; Phys denotes Physician.

Response alternatives are 1 = seldom or never, 2 = about once every 6 months, 3 = about once a month, and 4 = several times a month.

Physicians reported significantly more frequent use than the registered nurses on the following variables: *EBP use index*, *Formulate question*, *Search other sources*, and *Appraise research reports*. The physicians also reported more frequent use of EBP than the occupational therapists on the following variables: *Formulate question* and *Appraise research reports* (Table 4).

Only one significant difference was found between health professionals who had supervised students and those who had not. The supervisor group reported more frequent use of *Appraise research reports* than those who did not supervise students (m = 1.9 vs. m = 1.4; p = 0.001) (S3 Table).

### EBP capability beliefs index, EBP use index and years in profession among health professionals

We examined the relationships between EBP capability beliefs index, EBP use index and years in profession among the health professionals. The only significant relationship was found between the two EBP indices demonstrating that high capability beliefs were associated with more frequent use of EBP (Table 5).

**Table 5:** Relationships between evidence-based practice capability beliefs index, evidence-based practice use index and years in profession among health professionals.

	EBP capability beliefs index	EBP use index	Years in professions
**EBP capability beliefs index**	1	*r* = 0.597; p <0.001	*r* = -0.125; p = 0.214
**EBP use index**	*r* = 0.597; p <0.001	1	*r* = -0.005; p = 0.958
**Years in profession**	*r* = -0.125; p = 0.214	*r* = -0.005; p = 0.958	1

Pearson Correlation Coefficient test.

EBP denotes evidence-based practice.

## Discussion

This study highlights some interesting findings on similarities and differences in capability beliefs to perform EBP among multi-professional health professionals and students in a geriatric setting.

### Health professionals and students self-reported capability beliefs on EBP

High capability beliefs on EBP were identified in the student group and to some extent in the health professional group. The students rated significantly higher capability beliefs than health professionals on *EBP capability beliefs index*, and on *formulate questions* and *appraise research reports*. In contrast, the health professionals rated significantly higher capability beliefs on *search other sources*.

In a previous study a cut off for high capability beliefs has been set at >7 on the *EBP capability beliefs index* using the EBPCBS [12]. Health professionals in this study reported in general above this threshold value. Similar results have been reported in a smaller intervention study from the United States in a group of registered nurses, physical therapists, pharmacists and social workers working in an acute care hospital, in which the respondents report a mean value of 7.3 on the EBP capability beliefs index prior to a journal club intervention and a mean value of 7.8 after the intervention [35]. In our study the health professionals reported some variations between their capability beliefs for the different EBP activities, showing lower mean values for *formulate question* and *appraise research reports* indicating less confidence to perform these two EBP activities. However, no significant differences were identified between the four health professional groups regarding their capability beliefs on EBP. This is an important finding as high EBP capability beliefs has been shown to be strongly associated with the self-reported use of EBP [12,36].

The students reported high capability beliefs (mean values >8) on the four EBP activities *Formulate question*, *Search databases*, *Search other sources* and *Appraise research reports* which could be related to the steps in the research process. The Swedish Higher Education Act has over the past ten years emphasized the importance of anchoring higher education to relevant research aiming for all undergraduate students to learn to critical appraise scientific articles, identify and solve problems using the research process [37]. Thus, according to our findings the university and university colleges seem to prepare the students, at least in this context, with the confidence to accomplish the first four activities in the EBP process.

The students rated lower capability beliefs on the two EBP activities *Implement knowledge* and *Evaluate practice* although the mean values for these two activities were >7 indicating high capability beliefs. In a national study by Florin and coworkers [19] in a group of 1440 nursing students from 26 Swedish universities the respondents report a mean value of 8.1 on the EBP capability beliefs index and between 7.5 and 8.9 on the six EBP activities. As in our study the students rate the lowest mean values on the two EBP activities *Implement knowledge* and *Evaluate practice*. Florin and coworkers [19] have also investigated whether there were any differences between the students’ capability beliefs depending on which of the 26 universities they were studying. Significant differences are presented between the student groups from the 26 universities on the EBP capability beliefs index and the first four EBP activities, but no significant differences are presented between the student groups from the 26 universities regarding the EBP activities on *Implement knowledge* and *Evaluate practice*. Furthermore, the nursing students in the study by Florin and coworkers [19] have been asked about their experiences of educational support for research utilization in campus education and in clinical education. The students report less support during clinical education compared to campus education, and no differences between the universities in regards to this question are presented [19]. There seems to be a need to develop and enhance the support to the students during the clinical education, particularly to improve the students’ capability beliefs on implementing and evaluating new knowledge into practice.

We could not identify any significant differences between students from the four programs regarding their ratings on the EBP capability beliefs index or the six EBP activities which indicates that the students, regardless of program, reported to have the capability beliefs to use EBP. The senior students rated higher capability beliefs than the novice students on *Search databases* which seems logical as the senior students have had longer time of exposure and training on this specific task. Furthermore, students who stayed at the clinical placement for a longer period (three weeks and longer) scored higher on the EBP activity to *Search other sources* than students with shorter period. This activity includes asking colleagues, and it seems reasonable that a longer clinical placement at the unit may provide students more opportunity to ask supervisors and other staff for information on various topics. With the terminology of Dall’Alba and Sandberg [24] we argue that students with longer placements have more time to develop an embodied understanding of practice, e.g. on how to search other sources in such a practice.

It is interesting to note that the students in our study rated significantly higher on the EBP capability beliefs index than the health professionals, indicating a greater ability to perform the EBP process at large. In particular, the students reported higher capability beliefs on the two EBP activities *Formulate questions* and *Appraise research reports* than the health professionals. As earlier discussed, the universities have improved teaching on the research process and the content in the syllabus has to be scientifically founded. The students have been exposed from the first semester of their programs to formulate questions, and to critically appraise and compile literature and other information used in their learning activities and exams. However, the health professionals rated higher capability beliefs on the EBP activity *Search other sources* than the students which includes asking colleagues and peers. Previous research has recurrently identified that health professionals often use colleagues and peers as information sources for various decisions in practice [4,38]. However, seeking information from colleagues and peers will not obviously increase the evidence-based knowledge.

### Health professionals’ use of EBP

On the question of use of EBP among health professionals in clinical practice, our study found an area of improvement, particularly among registered nurses and occupational therapists. There were great variations on how often the different activities were completed. The EBP activity *Search other sources* was performed several times per month while *Appraise research reports* was performed seldom or once every six months. Previous studies investigating registered nurses, using the EBPCBS, have reported similar results on the use of the various EBP activities, indicating more frequent use of *Search other sources* and less frequent use of *Formulate question* and *Appraise research reports* [32,33].

One unanticipated finding was the differences between the professional groups in reported use of EBP activities. The physicians reported more frequent use of the EBP activities *Formulate questions*, *Search other sources* and *Appraise research reports* than registered nurses and occupational therapists. This was to some extent surprising as we did not identify any differences between their ratings on capability beliefs regarding EBP. We can only speculate on plausible reasons. The factor EBP capability beliefs is only one among many other essential factors for successful implementation of evidence into clinical practice. The PARIHS framework highlights the importance of a supportive context and access for facilitation for practitioners to enhance the uptake of evidence [11]. A supportive context consists of supportive leadership, change-friendly culture and access to resources according to the PARIHS framework. As our study was performed at a single center, one assumption could be that the context and culture could be perceived equally over professional borders. However, at the department there are different front-line managers for the different professional groups (physicians, OT/PT and nurses). These managers might have varied knowledge, skills and attitudes towards EBP. Thereby there may be various sub-cultures and traditions in the professional groups. Furthermore, there might be differences between the professional groups in regard to resources such as access to seminars and courses, and time to participate in these.

Further reasons for the differences between the professional groups’ use of EBP activities could be found in the review by Rousseau and Gunia [39] about the psychology of EBP implementation. The authors summarize their findings that there are three key contributors to implement EBP: practitioner ability, motivation, and opportunity to practice. To be able to implement evidence into practice, the clinician needs to have the ability and the motivation to practice EBP but also the opportunity to practice EBP in the daily work. Depending on the clinical situation patients may have immediate needs and problems which health professionals instantly have to solve. In these situations, health professionals might not formulate questions, search for evidence and appraise research reports; instead health professionals might act according to existing guidelines to quickly direct them to use adequate interventions or treatments. However, if these guidelines are not updated according to scientific evidence, there may be a risk that they are outdated and, even worse, incorrect. In units where many of the patients have immediate needs, the front-line managers should have the responsibility to arrange meetings and seminars for health professionals to have the opportunity to formulate questions, appraise research reports and update their knowledge and guidelines. Therefore, it is important to reflect on the activities in the EBP-process when working in an acute as well as in a non-acute clinical situation. In our study we did not collect data on contextual factors such as the health professionals’ perceptions on the context, e.g., supportive leadership and workplace culture or resources of various types, and their views on what support or hinder them to more frequently use EBP. Consequently, we are not able to explain why there were differences between health professional groups.

The health professionals who supervised students reported significantly more frequent use of *Appraise research reports* than those who did not supervise students. One explanation could be that the students discuss the evidence base with their supervisors when completing learning activities during the working place period. A qualitative study based on focus group interviews with physiotherapy students, supervisors and clinical teachers support that EBP discussions are common [20]. However, the students also need a lot of time and guidance on how to perform clinical skills [20]. Another explanation could be that health professionals who are more involved in EBP and, in particular, the appraisal of research reports, are more interested in being supervisors for students in clinical practice. A note of caution is due here since we only have studied health professionals in a geriatric setting at a single center. Nevertheless, the findings need to be further explored.

### Strength and limitations

In this study, we used the instrument EBPCBS to assess capability beliefs on EBP and self-reported use of EBP. Among 24 validated instruments, the EBPCBS has been found to have the highest validity and to be the most practical instrument to assess evidence-based knowledge, skills and attitudes in nursing practice [40]. The instrument EBPCBS has mostly been used to collect data on self-reported capability beliefs among nurses and nursing students [19,34,35]. There is one study from the United States which has used the EBPCBS in a group of registered nurses, physical therapists, pharmacists and social workers [35]. In the present study we used the instrument also among occupational therapists, physicians, physiotherapists and their corresponding students which may be considered a limitation. The questions in the EBPCBS may be perceived in different ways depending on profession. Professions with a long academic history might perceive the questions in another way than professions with a short academic background, e.g., *Search databases* could mean PubMed for some, and Google Scholar for others. Furthermore, the self-reported nature of the instrument limits its validity. It may be difficult for health professionals to distinguish to what extent their clinical decisions in practice are grounded on scientific evidence, on consensus knowledge, or on their own clinical experience. This may result in health professionals’ overestimation or underestimation of their use of evidence in practice. Also for the students it may be difficult to evaluate their capability beliefs on the EBP activities, and therefore the students might have overestimated their ability for the EBP activities. Given the nature of the instrument the use of parametric statistics may be criticized. However, sensitivity analysis showed similar results using non parametric statistics. Due to the small sample size we did not conduct any regression analyses. Therefore, we cannot say whether, for example, longer or shorter workplace learning is independently associated with EBP capability beliefs.

The overall response-rate (80% for health professionals and 73% for students) was acceptable and most questions within the questionnaire were answered by the health professionals (96%) and the students (97%). The study population was, however, rather small and recruited from one setting only, and consequently the generalizability is limited. There is a need for future research, preferably conducted as multi-center studies. Furthermore there is a need for studies including measures on contextual factors to be able to explore potential differences in organizational conditions to apply EBP between different health professional groups.

## Conclusions

This paper has examined comparisons of self-reported capability beliefs on EBP between health professional and student groups, and comparisons of the use of EBP between four health professional groups in a hospital-based acute geriatric care setting in Sweden. Identified similarities and difference in capability beliefs and use of EBP have several clinical implications. An association between EBP capability beliefs and use of EBP was identified in-line with Bandura’s self-efficacy theories [12]. This study shows that there is an obvious need for a more frequent use of EBP in acute geriatric care of older patients, particularly among registered nurses and occupational therapists. Interprofessional teamwork is essential for good quality care for the patient population served. To accomplish EBP in this team based context, our findings suggest that management initiatives and strategies are needed.

Our finding that clinical supervisors for students more frequently appraise research reports as compared to other health professionals is of importance. To interact with students while conducting patient care in a geriatric setting may contribute to motivate health professionals to base clinical decisions on evidence and thereby contribute to EBP in routine care. We hypothesis that the high EBP capability beliefs to formulate questions and appraise research reports among students may inspire staff and contribute to an increased use of these EBP activities in daily practice. However, further studies are needed to test this hypothesis.

Finally, undergraduate education for future health professionals needs to focus more on learning activities during workplace learning for students to practice implementation and evaluation of new knowledge.

## Supporting information

S1 Appendix The Evidence-Based Practice Capability Beliefs Scale (EBPCBS). (DOC)

S1 Table Comparison of reported capability beliefs on evidence-based practice between supervisors and non-supervisors. (DOCX)

S2 Table Comparisons of reported capability beliefs on evidence-based practice among novice and senior students, and students with shorter and longer clinical placement. (DOCX)

S3 Table Comparison of reported use of evidence-based practice between supervisors and non-supervisors. (DOCX)

S1 Dataset EBP_Health_professionals_Students_Bostrom.sav. (SAV)
