# Supplementary material for: Capability beliefs on, and use of evidence-based practice among four health professional and student groups in geriatric care: A cross sectional study
Source: PLoS One. 2018 Feb 14;13(2):e0192017. doi: 10.1371/journal.pone.0192017 (PMC5812600; doi:10.1371/journal.pone.0192017)
Supplement: S1 Appendix — (DOC) [file pone.0192017.s001.doc]

**S1 Appendix. The Evidence-Based Practice Capability Beliefs Scale (EBPCBS).**

| **1.** | **Rate your *performance* in the following tasks.** *(to health professionals and students)* | | | | | | | | | | | | | | | | |
| --- | --- | --- | --- | --- | --- | --- | --- | --- | --- | --- | --- | --- | --- | --- | --- | --- | --- |
|  | *Please mark one option on each line with a cross (x).* | **I’m sure I can manage that** | | | | | **I might manage that** | | | | | | **No, I can’t manage that** | | | | |
|  |  | | 10 | | 9 | 8 | 7 | | 6 | 5 | 4 | | 3 | 2 | 1 | | 0 |
| a. | Formulating questions about clinical practice to search for new research-based knowledge. | |  | |  |  |  | |  |  |  | |  |  |  | |  |
| b. | Using databases to search for knowledge. | |  | |  |  |  | |  |  |  | |  |  |  | |  |
| c. | Using other information sources  (e.g. books, journals or asking colleagues). | |  | |  |  |  | |  |  |  | |  |  |  | |  |
| d. | Appraising research reports. | |  | |  |  |  | |  |  |  | |  |  |  | |  |
| e. | Contributing to change in clinical practice by implementing research knowledge. | |  | |  |  |  | |  |  |  | |  |  |  | |  |
| f. | Participating in evaluating whether clinical practice is based on research knowledge. | |  | |  |  |  | |  |  |  | |  |  |  | |  |
| **2.** | **How often do you *carry out* the following tasks in your work?** *(to health professionals)* | | | | | | | | | | | | | | | | |
|  | *Please mark one option on each line with a cross (x).* | | | **Rarely/**  **Never** | | | | **About once every six months** | | | | **About once a month** | | | | **Several times a month** | |
|  |  | | | 1 | | | | 2 | | | | 3 | | | | 4 | |
| a. | Formulating questions about clinical practice to search for new research-based knowledge. | | |  | | | |  | | | |  | | | |  | |
| b. | Using databases to search for knowledge. | | |  | | | |  | | | |  | | | |  | |
| c. | Using other information sources  (e.g. books, journals or asking colleagues). | | |  | | | |  | | | |  | | | |  | |
| d. | Appraising research reports. | | |  | | | |  | | | |  | | | |  | |
| e. | Contributing to change in clinical practice by implementing research knowledge. | | |  | | | |  | | | |  | | | |  | |
| f. | Participating in evaluating whether clinical practice is based on research knowledge. | | |  | | | |  | | | |  | | | |  | |
