# Supplementary material for: Capability beliefs on, and use of evidence-based practice among four health professional and student groups in geriatric care: A cross sectional study
Source: PLoS One. 2018 Feb 14;13(2):e0192017. doi: 10.1371/journal.pone.0192017 (PMC5812600; doi:10.1371/journal.pone.0192017)
Supplement: S1 Table — (DOCX) [file pone.0192017.s002.docx]

**S1 Table. Comparison of reported capability beliefs on evidence-based practice between supervisors and non-supervisors.**

|  | Supervisors | Non-supervisors | P-value |
| --- | --- | --- | --- |
| EBP capability beliefs index | 7.7 (1.6) | 7.3 (1.8) | 0.260 |
| Formulate questions | 7.3 (2.0) | 6.4 (2.6) | 0.056 |
| Search databases | 7.9 (2.2) | 7.8 (2.5) | 0.866 |
| Search other sources | 9.1 (1.4) | 9.3 (1.0) | 0.567 |
| Appraise research reports | 7.1 (2.4) | 6.4 (2.4) | 0.200 |
| Implement knowledge | 7.4 (2.2) | 7.3 (2.2) | 0.794 |
| Evaluate practice | 7.4 (1.9) | 6.7 (2.6) | 0.136 |

Values are given as mean ± standard deviation (SD). The p-values are calculated by unpaired t-test between supervisors and non-supervisors in the health professional group.

Response alternatives range from 0 (No, I can’t manage that) to 10 (I’m sure I can manage that).

EBP denotes evidence-based practice.
