# Supplementary material for: Capability beliefs on, and use of evidence-based practice among four health professional and student groups in geriatric care: A cross sectional study
Source: PLoS One. 2018 Feb 14;13(2):e0192017. doi: 10.1371/journal.pone.0192017 (PMC5812600; doi:10.1371/journal.pone.0192017)
Supplement: S2 Table — (DOCX) [file pone.0192017.s003.docx]

**S2 Table. Comparisons of reported capability beliefs on evidence-based practice among novice and senior students, and students with shorter and longer clinical placement.**

|  | Novice student | Senior student | P-value | Shorter placement | Longer placement | P-value |
| --- | --- | --- | --- | --- | --- | --- |
| EBP capability beliefs index | 7.8 (1.5) | 8.2 (1.5) | 0.120 | 7.8 (1.6) | 8.2 (1.4) | 0.098 |
| Formulate questions | 7.8 (2.0) | 8.3 (1.8) | 0.153 | 8.0 (1.9) | 8.2 (1.9) | 0.593 |
| Search databases | 7.7 (2.0) | 8.8 (1.2) | 0.001 | 8.2 (1.5) | 8.4 (1.8) | 0.331 |
| Search other sources | 8.5 (1.7) | 9.0 (1.3) | 0.058 | 8.4 (1.7) | 9.0 (1.3) | 0.014 |
| Appraise research reports | 8.0 (1.8) | 8.4 (1.6) | 0.202 | 7.9 (1.9) | 8.4 (1.5) | 0.074 |
| Implement knowledge | 7.3 (1.9) | 7.5 (2.3) | 0.570 | 7.1 (2.0) | 7.6 (2.1) | 0.124 |
| Evaluate practice | 7.5 (1.8) | 7.4 (2.3) | 0.854 | 7.3 (2.1) | 7.6 (2.1) | 0.364 |

Values are given as mean ± standard deviation (SD). The p-values are calculated by unpaired t-test.

Response alternatives range from 0 (No, I can’t manage that) to 10 (I’m sure I can manage that).

EBP denotes evidence-based practice.
