# Supplementary material for: Capability beliefs on, and use of evidence-based practice among four health professional and student groups in geriatric care: A cross sectional study
Source: PLoS One. 2018 Feb 14;13(2):e0192017. doi: 10.1371/journal.pone.0192017 (PMC5812600; doi:10.1371/journal.pone.0192017)
Supplement: S3 Table — (DOCX) [file pone.0192017.s004.docx]

**S3 Table. Comparison of reported use of evidence-based practice between supervisors and non-supervisors.**

|  | Supervisors | Non- supervisors | P-value |
| --- | --- | --- | --- |
| EBP capability beliefs index | 2.5 (0.7) | 2.3 (0.6) | 0.142 |
| Formulate questions | 2.3 (1.1) | 2.0 (1.0) | 0.217 |
| Search databases | 2.7 (1.1) | 2.4 (1.1) | 0.129 |
| Search other sources | 3.7 (0.6) | 3.6 (0.6) | 0.790 |
| Appraise research reports | 1.9 (1.0) | 1.4 (0.6) | 0.001 |
| Implement knowledge | 2.5 (1.0) | 2.5 (1.0) | 0.806 |
| Evaluate practice | 2.1 (1.0) | 2.0 (1.1) | 0.611 |

Values are given as mean ± standard deviation (SD). The p-values are calculated by unpaired t-test.

Response alternatives are 1=seldom or never, 2=about once every 6 months, 3=about once a month and 4=several times a month.

EBP denotes evidence-based practice.
